# Supplementary figures and images for: Methanolic crude extract of Litsea Monopetala leaves combats oxidative stress, clot formation, inflammation and stool frequency in animal model
Source: PLoS One. 2025 May 27;20(5):e0313706. doi: 10.1371/journal.pone.0313706 (PMC12111300; doi:10.1371/journal.pone.0313706)

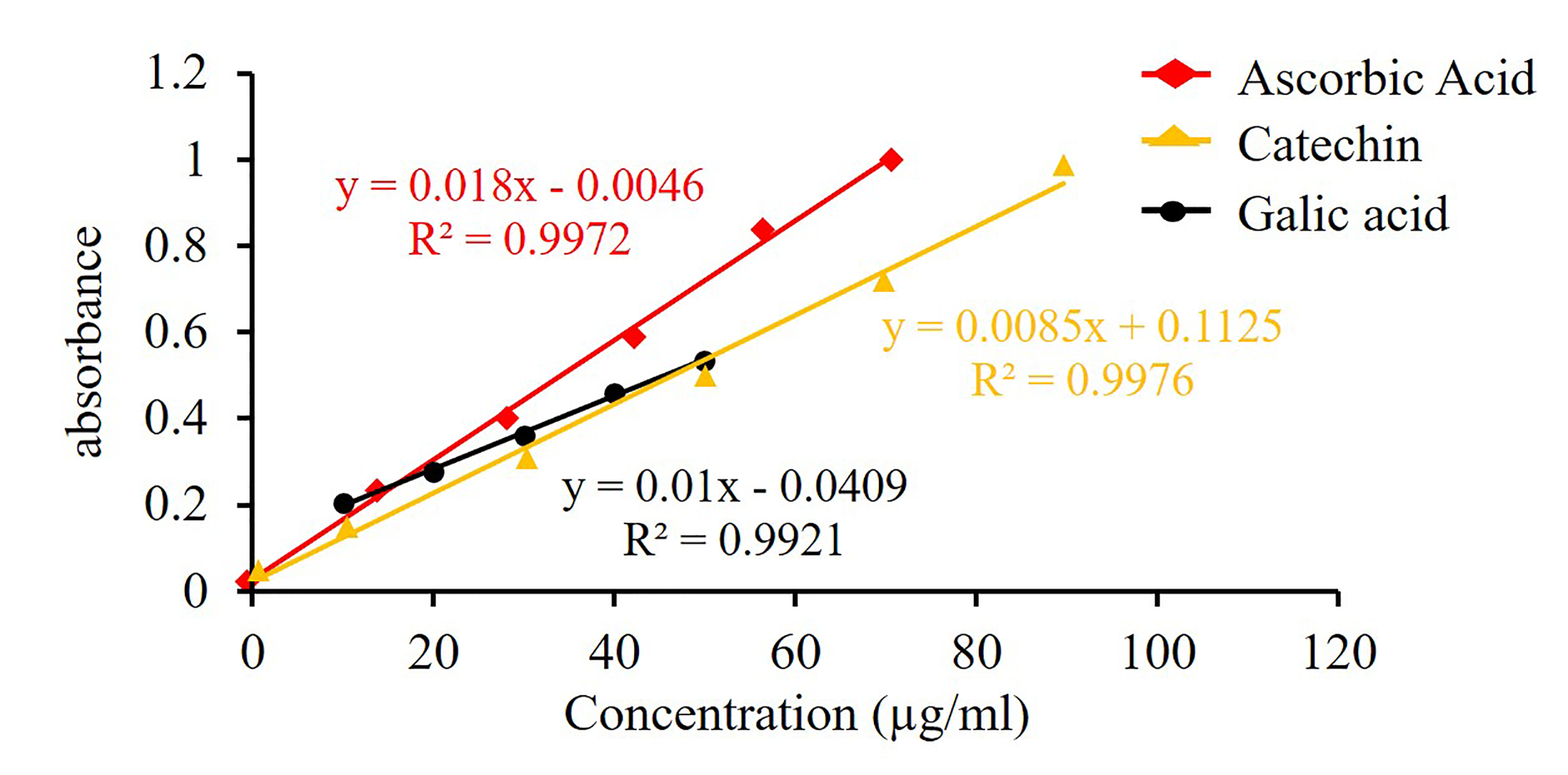

Supplement: S1 Fig — (TIF) [file pone.0313706.s001.tif]
