# Supplementary material for: Methanolic crude extract of Litsea Monopetala leaves combats oxidative stress, clot formation, inflammation and stool frequency in animal model
Source: PLoS One. 2025 May 27;20(5):e0313706. doi: 10.1371/journal.pone.0313706 (PMC12111300; doi:10.1371/journal.pone.0313706)
Supplement: Attachment — Submitted filename: Response to Reviewers.docx [file pone.0313706.s005.docx]

**Response to academic editor and reviewer’s comments**

We have addressed all issues indicated in the review report, and believed that the revised version can meet the journal publication requirements. The followings are our amendments to the reviewer’s comments written by point-by-point. We hope our present form of amended manuscript can meet with your standard as well as PLOS ONE.

**Comment from Editor**

1. Please ensure that your manuscript meets PLOS ONE's style requirements, including those for file naming. The PLOS ONE style templates can be found at <https://journals.plos.org/plosone/s/file?id=wjVg/PLOSOne_formatting_sample_main_body.pdf> and <https://journals.plos.org/plosone/s/file?id=ba62/PLOSOne_formatting_sample_title_authors_affiliations.pdf>.

*Response: Thank you for your comment. We believe the revised manuscript will meet the PLOS ONE’s Publication requirement.*

2. Please match your authorship list in your manuscript file and in the system.

*Response: Thank you for your comment. The authorship in the manuscript file and the system has been rechecked and made synchronous.*

3. In your Methods section, please provide additional information regarding the permits you obtained for the work. Please ensure you have included the full name of the authority that approved the field site access and, if no permits were required, a brief statement explaining why.

*Response:* *Thank you for your comment. This is not competing interests of data presented in this study (line 4457-458)*

4. To comply with PLOS ONE submissions requirements, in your Methods section, please provide additional information regarding the experiments involving animals and ensure you have included details on (1) methods of sacrifice, (2) methods of anesthesia and/or analgesia, and (3) efforts to alleviate suffering.

*Response: Thank you for your comment. Information regarding (1) methods of sacrifice, (2) methods of anesthesia and/or analgesia, and (3) efforts to alleviate suffering of the experiments animals has been discussed in detail in the materials and method section. Please have a look (line 128-142)*

5. We note that your Data Availability Statement is currently as follows: [All relevant data are within the manuscript and its Supporting Information files.]

*Response: Thank you for your comment. Data availability statement is followed as per PLOS ONE journal guideline. All relevant data are either within the manuscript or its supporting information files.*

**Comment from Reviewer #1:**

To access the antidiarrheal activity ethanolic extracts are reported in the results while the objective was to study crude methanolic extracts. This discrepancy needs to be clarified
Format of the references should be checked
References mentioned in the text should also be included in the bibliography

*Response:*

*Dear reviewer,*

*Thank you very much, and we appreciate your time and effort in reviewing our manuscript to improve. We addressed all the points and revised our manuscript accordingly. We are requesting you reconsider the manuscript.*

*We are expressing apology for the unintentional typographic mistakes and we corrected it in the revised manuscript. Anomalies in the reference format have been revised as per the journal's guidelines. The bibliography has also been revised as per references in-text citation. Please have a look (line 463-600)*

**Comment from Reviewer #2:**

The manuscript titled “Methanol Crude Extract of *Litsea Monopetala* Leaves combats oxidative stress, clot formation, inflammation, and stool frequency in Animal Model” presents interesting findings on the therapeutic potential of *Litsea monopetala*. However, there are several points that need to be addressed to improve the clarity, structure, and accuracy of the manuscript. Below are detailed suggestions for revision:

*Response:*

*Dear reviewer,*

*Thank you very much and we appreciate your time in reviewing the manuscript. We addressed all of your suggestions and revised our manuscript accordingly.*

1. Revised Sentence on Antioxidant Assessment: “To assess the antioxidant… phytochemical screening was performed to estimate the bioactive compounds (e.g.- phenol, flavonoid, alkaloids, tannins, and others) present.” This sentence should be revised to enhance clarity and precision.

*Response: Thank you for your comment. The sentence has been revised. Please have a look (line 28-34)*

*The revised texts are follows:*

We assessed the antioxidant activity using DPPH (2,2-diphenyl-1-picrylhydrazyl) free radical scavenging and total phenolic content tests, while thrombolytic activity was evaluated via clot lysis assays. The *in-vivo* analgesic and antidiarrheal activities were tested by two standard methods e.g., acetic acid-induced and castor oil-induced animal model, respectively. Prior to *in vivo* and *in vitro* evaluation of the pharmacological activities phytochemical screening was performed to estimate the bioactive compounds (e.g., phenol, carbohydrates, reducing sugars, tannins, alkaloids, flavonoids, saponins and steroids) present.

2. The abstract lacks a clear structure separating the introduction, methods, results, and conclusions. Please revise the abstract to follow the typical structure of scientific abstracts: introduction, material and methods, results and conclusions.

*Response: Thank you for your comment. The abstract has been revised as per your comment. Please have a look in the revised manuscript (line 23- 47)*

3. To date, no study conducted in in-vivo experiments to find possible therapeutic benefits for common health problems...” This statement is inaccurate as there are multiple published studies on the in vivo effects of Litsea monopetala. The authors should review the following studies and revise their statement accordingly. Please cite these references and modify the statement to accurately reflect the existing literature.

*Response: Thank you for your comment. We corrected the statement para in the introduction section as per your comment. Please have a look in the revised manuscript (line 87-93.)*

*The revised texts are follows:*

There have been reports of analgesic, antibacterial, antioxidant, antidiabetic, anti-diarrheal, anti-fungal, anti-arrhythmic and cytotoxic properties *LM* leaves extract [23-25]. Nasrin F., *et al* (2015) evaluated the antidiarrheal activity of ethanolic extracts from *LM* leaves and observed 60% inhibition of defecation at 400 mg/kg body weight dose [26]. *In vivo* analgesic, antiemetic and anxiolytic effect of methanolic extract of L. monopetala leaves was also evaluated in formalin induced animal model and demonstrated a statistically significant (P<0.05) reduction in paw licking [24]. Methanolic extracts of LM leaves were studied for antioxidant, analgesic and antidiarrheal properties [23]. This study showed antioxidant (*IC_5_*_0_ =223.22 μg/ml), analgesic (68.75% writhing inhibition) and antidiarrheal properties. In an *in-vitro* study, Lamichhane G *et al.* (2023) tested selected five plants, including *LM* leaves and observed remarkable antioxidant, antibacterial, anti-adipogenic, and anti-inflammatory activities from Nepal [27]. Thus, there is a strong rationale to validate the ethnomedicinal uses of *LM* leaves extract of Bangladesh origin.

DPPH assay is a common method that measures the *in-vitro* antioxidant capacity, while two standard methods acetic acid-induced and castor oil-induced animal model are also frequent to test *in-vivo* analgesic and antidiarrheal activities, respectively [23-27]. Thrombolytic effects are typically assessed through in vitro testing with human blood by measuring the percentage of the clot lysis [23-27]. In this study, we have attempted to evaluate not only the *in-vitro* antioxidant and thrombolytic, but also *in-vivo* analgesic and antidiarrheal properties of *LM* leaves in different extracts. This research would be a valuable data addition to the effective and safe use of LM Plant in the ethnomedicinal arena.

a. In vivo antidiarrheal study of ethanolic extracts of Mikania cordata and Litsea monopetala leaves DOI: 10.3329/bjp.v10i3.23402

*We revised and added the reference. Please have a look in the revised manuscript (ref no 26, line 540-541)*

b. Evaluation of in vivo analgesic, antiemetic and anxiolytic effect of methanolic extract of Litsea monopetala in animal model. DOI:10.15562/phytomedicine.2019.102

*We revised and added the reference. Please have a look in the revised manuscript (ref no 24, line 534-536)*

c. The Analgesic Potential of Litsea Species: A Systematic Review. doi: 10.3390/molecules29092079

*We revised and added the reference. Please have a look in the revised manuscript (ref no 18, line 511-513)*

d. Evaluation of Antioxidant, Analgesic and Antidiarrheal Activities of Methanolic Extract of Litsea monopetala (roxb.) Leaves DOI: 10.4172/2167-065X.1000185

*We revised and added the reference. Please have a look in the revised manuscript (ref no 23, line 531-533)*

4. Figures 1, 3, and 4 are not clear due to low resolution. Authors need to improve the resolution of these figures to ensure they are of sufficient quality for publication. Higher-resolution images will allow for clearer interpretation of the data.

*Response: Thanks a lot. Figures 1, 3, and 4 have been revised and resubmitted through the system*

5. Figure 2: Move the standard curves for Gallic Acid, Catechin, and Ascorbic Acid to the supplementary data section. This will streamline the main manuscript while still providing essential data for reference.

*Response: Thanks for your suggestion. We moved figure 2 in the supplementary data section (S1_Fig.) and resubmitted through the system.*

6. Authors should ensure that the unit of expression for results is consistent throughout the manuscript. Please decide whether to use "µg/mL" or "µg/ml" and apply it uniformly across all sections, tables, and figures.

*Response*: *Thank you very much for pointing out this mistake. As per your comment, we revised our manuscript with “*µg/ml” *to improve infirmity in the manuscript. Please have a look in the revised manuscript (Fig 2, Fig 3, line 37, 221, 226-228)*

7. Table 1: The remarks represented by "+" and "–" signs should be clarified. Include a detailed explanation of what each symbol represents in the table's footnotes to improve

*Response: Thanks for the comment. The manuscript has been revised and remarks has been clarified with footnotes. Please have a look in the revised manuscript. (line no 632)*

8. A comprehensive revision of the manuscript is necessary to improve the grammar, sentence structure, and overall readability. Consider using a professional language editing service or revising the manuscript thoroughly to ensure it meets the publication standards

*Response: Thanks for the comment. The manuscript has been revised and the language has been checked*. *Please have a look in the revised manuscript*

Sincerely

**Sarder Arifuzzaman, M.S.**

Sr. Lecturer

Department of Pharmacy

World University of Bangladesh

Avenue 6 and Lake Drive Road, Sector#17, Uttara, Dhaka-1230, Bangladesh

C.P.: +8801776883100

Email: [sarder.arifuzzaman@pharmacy.wub.edu.bd](mailto:sarder.arifuzzaman@pharmacy.wub.edu.bd)

arifpharmju@gmail.com
